# Performance of the school-based human papillomavirus vaccine uptake in Tshwane, South Africa

**DOI:** 10.4102/sajid.v38i1.492

**Published:** 2023-01-31

**Authors:** Tladi D. Ledibane, Neo R. Ledibane, Moliehi Matlala

**Affiliations:** 1 Department of Public Health Medicine, School of Medicine, Sefako Makgatho Health Sciences University, Pretoria, South Africa; 2 School of Health Systems and Public Health, Faculty of Health Sciences, University of Pretoria, Pretoria, South Africa; 3 Department of Public Health Pharmacy Management, School of Pharmacy, Sefako Makgatho Health Sciences University, Pretoria, South Africa

**Keywords:** human papillomavirus vaccine, vaccine uptake, school-based vaccination programme, Tshwane, South Africa

## Abstract

**Background:**

Human papillomavirus (HPV) vaccine is an effective preventive measure against HPV infection and HPV-associated cervical cancer. South Africa introduced its HPV vaccination programme in 2014.

**Objectives:**

The authors assessed the uptake of HPV vaccine in the school-based HPV vaccination programme in Tshwane Health District for the year 2019 and compared the vaccine uptake (VU) between fee-paying and no-fee public schools.

**Method:**

The study method was cross-sectional, using routine electronic health records of the HPV vaccination programme. The study population included all Grade 4 school-girls between the ages of 9 and 14 years who attended public schools in 2019 in the Tshwane Health District.

**Results:**

The pooled VU for the Tshwane Health District was 72.0%, considerably lower than the target of 80.0%. The number of girls who received dose one and dose two in 2019 was 16 122 (73.0%) and 15 734 (71.0%), respectively, excluding the catch-up figures. In addition, 82.2% of fee-paying schools achieved VU of above 80% versus 65.5% of no-fee schools (*p* = 0.022).

**Conclusion:**

The lower than target levels of VU for HPV among girls in Tshwane Health District, particularly in those attending no-fee schools, is concerning. Interventions should be adopted to optimise programme performance so as to achieve the target VU of 80%.

**Contribution:**

This study showed the need to strengthen sensitisation and social mobilisation efforts, particularly among no-fee schools to improve the VU.

## Introduction

Cervical cancer is associated with human papillomavirus (HPV) infection,^1^ making HPV an important public health pathogen. Human papillomavirus is a common, sexually transmitted infection acquired shortly after sexual debut.^1^ Human papillomavirus infection accounts for nearly 90% of cervical cancer cases.^2^ There are over 100 HPV serotypes, most of which cause self-limiting infection which resolves within 2 years.^2^ About 14 HPV serotypes are associated with cervical cancer.^2^ Human papillomavirus 16 and 18 serotypes are considered significant oncogenic types and account for more than 70% of cervical cancers globally.^3,4^ Nononcogenic HPV serotypes can cause diseases such as genital warts and respiratory papillomatosis.^4^

Globally, cervical cancer affects more than 570 000 women annually, with 311 000 cervical cancer–associated deaths annually,^2,5^ making it the fourth most typical cause of cancer deaths among women worldwide.^5^ Low- to middle-income countries (LMICs) contribute more than 88% to the global burden of cervical cancer.^5,6,7^ Sub-Saharan Africa (SSA), Eastern Europe and Western Asia have the highest incidence and mortality of cervical cancer globally.^5,7^

Gardasil^®^ and Cervarix^®^ HPV vaccines were licenced in 2006 after showing high levels of efficacy and a good safety profile.^8^ Cervarix^®^ is a bivalent (bHPV – 16, 18) vaccine, whereas Gardasil^®^ is a quadrivalent (qHPV – 6, 11, 16, 18) and Gardasil 9^®^ is a nonavalent (nHPV – 6, 11, 16, 18, 31, 33, 45, 52, 58) vaccine. All HPV vaccines are subunit vaccines produced from a single virion that forms virus-like particles (VLP) but without HPV DNA. Thus, they are neither infectious nor oncogenic. Virus-like particles are highly immunogenic and elicit higher antibody titres than natural infection.^9^ The correlate of protection of HPV vaccines is thought to be the neutralising antibodies. Both vaccines contain an aluminium salt adjuvant that precipitates the VLPs.^10,11^

Despite the benefits of HPV vaccinations, vaccination rates in many LMICs remain low.^12^ The cost of vaccines, delivery cost and complexities for the target population are the key barriers^13^ to vaccine uptake (VU). For example, as of 2020, only 55% of World Health Organization (WHO)-affiliated countries had implemented the HPV vaccine rollout in their national immunisation programmes,^12^ most of which belong to high-income countries (HICs). Only 31% of African countries have implemented the HPV vaccine rollout in their national immunisation programmes, compared with 77% and 85% of countries in Europe and the Americas, respectively.^12^ The low coverage combined with the slow pace of HPV vaccine rollout and lack of access to HPV vaccines has resulted in low global coverage of 15%,^12^ thus a low reduction of the disease burden associated with HPV infection. The mean VU rate of HPV programmes achieves 68% for the first and 53% for the last dose of HPV,^12^ which is lower than the 90% uptake required to achieve herd immunity for HPV.^14^

School-based HPV vaccination programmes are more cost-effective and likely to achieve higher coverage than health facility–based programmes in LMICs.^15,16,17^ In countries that adopted the school-based strategy, HPV vaccine coverage was about 85% in LMICs,^15,16,17^ whereas the health facility-based HPV vaccine strategy yielded a coverage of about 50%.^15,16,17^ Furthermore, school-based programmes achieve a lower dropout rate of 7% between the first and second dose compared with 11% at facility-based programmes.^15,16,17^ Data from South Africa and other African countries employing the school-based approach reveal high HPV vaccine coverage^18,19^ compared with the facility-based approach.

The South African HPV vaccine programme, initiated in 2014, is a school-based programme targeted at girls aged 9 years or older in Grade 4 in public schools.^20^ The South African HPV vaccination programme provides a two-dose series of Cervarix® at 6-month intervals to girls over 9 years in Grade 4. However, concerns remain regarding the performance of the school-based HPV vaccination programme targeted at adolescent girls. Again, the HPV vaccination programme differs from the rest of South Africa’s extended immunisation programme, which is based at health facilities and targeted at children younger than six. As a result, the performance of the HPV vaccination programme may differ from health facility–based programmes. Furthermore, there is a paucity of data on the performance of the HPV vaccination programme in South Africa and the Tshwane health district.

The authors assessed the performance of the school-based HPV vaccination programme in the Tshwane Health District from 01 January 2019 to 31 December 2019. The objectives of the study were to: (1) assess the performance of the HPV vaccine in the school-based HPV vaccination programme in Tshwane, stratified by the fee-paying status of the schools, (2) assess the HPV vaccination programme performance and (3) report on the reasons for failing to vaccinate eligible girls during the vaccination campaign.

## Methods

### Description of the vaccination programme

The HPV vaccination programme is school based, with campaigns each year in March and September. Tshwane Health District hires additional staff for the duration of each campaign and begins planning activities such as microplanning, social marketing and sensitisation in advance of each campaign. During the planning phase of the campaigns, the school health team educates parents and girls about the benefits of vaccination against HPV. Vaccination teams visit each school once during each campaign. The vaccination teams administer missed doses during the next cycle of the vaccination campaign. The programme is centrally managed, with the budget allocated to the Tshwane Health District Office. Vaccines and other supplies are purchased through the office and are distributed to subdistricts.

### Context

**FIGURE 1:**
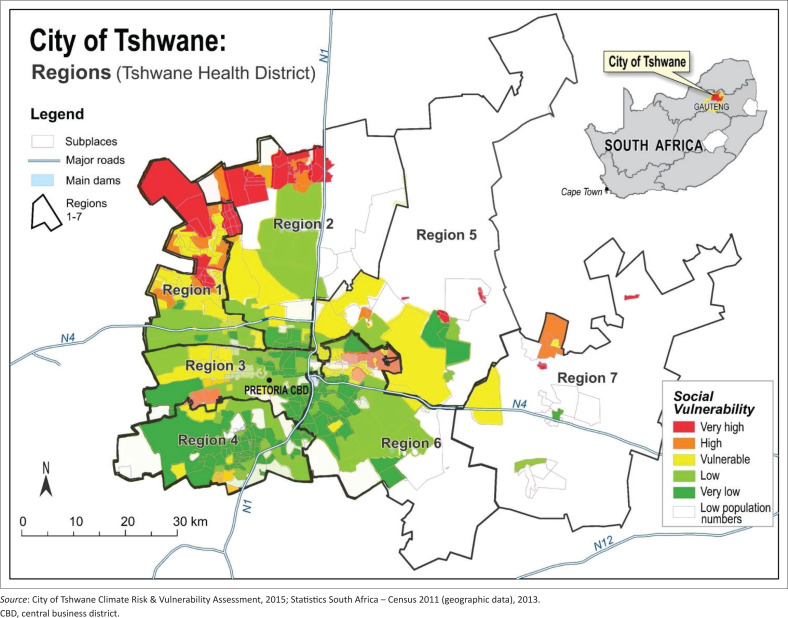
Tshwane Health District’s seven subdistricts, portraying the social vulnerability index.

The City of Tshwane is one of eight metropolitan municipalities in South Africa, with a population of approximately 2.92m.^21^ The Tshwane Health District is the largest municipality by surface area in South Africa and is divided into seven sub-districts (Figure 1). A social vulnerability index was developed by the Subplace Spatial Entity for Tshwane. Various social factors were taken into account, such as the number of informal settlements, overcrowding of households, female or child headed households, adult education rates, scale of poverty, population density, dependency ratio and unemployment rate. Regions 1, 2, 3 and 6 have very high social vulnerability index.^22^

South African public schools are categorised as either fee-paying or non-fee schools based on the social vulnerability status of the area where the school is situated. Fee-paying schools are concentrated in areas with a low social vulnerability index (Regions 3, 4, and parts of Regions 6 and 7). No-fee schools are situated in areas with a high social vulnerability index.

### Study design

The authors performed a cross-sectional study of the HPV vaccination programme through a retrospective review of electronic health records in the Tshwane Health District. All 360 public schools that offered school-based HPV vaccination in 2019 were included in the study. All electronic records of Grade 4 school-girls aged 9 to 14 years who attended public schools in Tshwane in 2019 formed part of the study population. No sampling was performed, and all eligible participant records were included for analysis.

### Data management and analysis plan

The HPV vaccination programme monitoring database served as the data source. The following variables were measured: the number of girls vaccinated per school during each campaign, the fee-paying status and name of each school, the subdistrict or region, the number of girls eligible for vaccination per school and reasons for missing vaccination during the school visit. Data regarding the fee paying status of the schools and the number of girls targeted for vaccination were obtained from the Department of Basic Education. Data were exported to Stata version 15 (StataCorp LLC, College Station, Texas, United States) for cleaning and analysis.

### Statistical analysis plan

Human papillomavirus vaccination performance indicators were assessed using VU, which is defined as the proportion of the target population that received the full course (two doses) during the campaign. The χ² test was applied to compare proportions, for example, VU stratified by the fee-paying status of the schools. The authors used one-way analysis of variance (ANOVA) to assess the differences in VU within and between subdistricts. *P*-values were considered significant if less than 0.05.

## Results

All 360 public schools in the Tshwane Health District participated in the HPV vaccination programme in 2019. There were 157 fee-paying schools (44%) and 203 no-fee schools (56%). The target population was 22 057 girls. Excluding catch-up figures, the number of girls who received doses one and two in the 2019 Grade 4 cohort was 16 122 (73%) and 15 734 (71%), respectively.

Table 1 depicts the number of girls in Grade 4, as well as VU, across Tshwane’s subdistricts. Subdistrict 1 (Region 1) had the highest number of girls at 6976, and Subdistrict 5 (Region 5) had the lowest enrolments at 784. The mean VU for the entire Tshwane Health District was 72%. The highest average VU was observed in Subdistricts 1 and 4 (Regions 1 and 4: 76.4% and 74.3%, respectively). Subdistrict 5 (Region 5) performed the least favourable, at 62.5%.

**TABLE 1:** Vaccine uptake among Grade 4 girls in Tshwane’s subdistricts (*N* = 22 057).

Subdistricts or regions	Number of girls in Grade 4	Percentage and number of girls vaccinated per subdistrict	
		%	*n*
1	6976	76.4	5330
2	3011	69.4	2090
3	4428	65.9	2918
4	2082	74.3	1547
5	784	62.5	490
6	3685	72.9	2686
7	1091	63.8	696

**Total**	**22 057**	**72.0**	**15 881**

Eighty-two percent (129) of fee-paying schools achieved the set target of VU (above 80%), whereas only 65% of no-fee schools achieved the set target, *p* = 0.022 (Table 2). The differences in VU between fee-paying and no-fee schools in all subdistricts were only statistically significant in Subdistrict 5; *p* = 0.026 (Table 3).

**TABLE 2:** Proportion of vaccine uptake among Grade 4 girls stratified by subdistricts and fee-paying status.

Subdistricts or regions	School fee-paying status		*p*
	Fee-paying VU	No-fee VU	
1	72.6	80.2	0.604
2	67.6	71.1	0.482
3	68.1	63.6	0.760
4	75.5	73.1	0.847
5	68.8	56.2	0.026
6	75.4	70.4	0.824
7	67.6	60.0	0.636

**Total**	**68.8**	**78.8**	**-**

VU, vaccine uptake.

**TABLE 3:** Tshwane’s sub-district VUR stratified by the school’s fee-paying status.

Fee-paying status	Uptake < 80%		Uptake ≥ 80%		Total
	Number of schools	%	Number of schools	%	
Yes	28	17.8	129	82.2	157
No	70	34.5	133	65.5	203

**Total**	**98**	**27.2**	**262**	**72.8**	**360**

### Reasons for not vaccinating during the campaign visits

**FIGURE 2:**
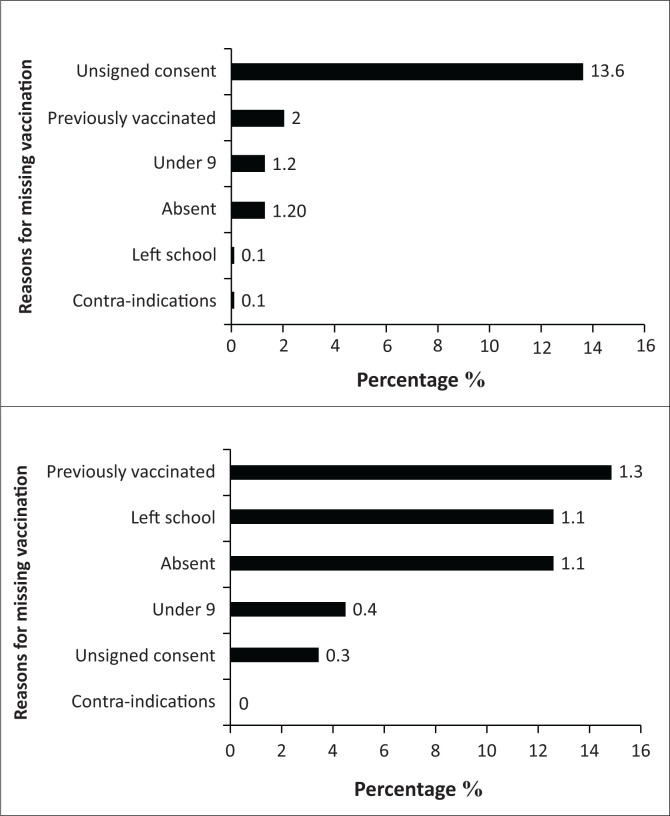
The proportion and reasons for missing vaccination during the March (a) and September (b) campaigns (*N* = 22 057).

Unsigned consent forms on the campaign day accounted for 13.6% and 1.2% of the girls not being vaccinated in the first and second rounds, respectively. Absenteeism accounted for 1.2% and 1.3% of the target population not being vaccinated in the first and second campaigns (Figure 2).

## Discussion

These findings shed light on the factors influencing the performance of the HPV vaccination programme in the Tshwane Health District and its subdistricts in South Africa. The VU in all but one subdistrict (Region 7) was below 80%, with no difference in the VU between fee-paying and no-fee schools. These findings are similar to those from studies conducted in SSA and KwaZulu-Natal.^12,20^ These previous studies show that school-based HPV vaccination programmes achieve higher VU than the facility-based vaccination programmes, a finding which was not analysed in this study as data were obtained from the school-based programme only.

The VU was reasonably high on aggregate (72.0%) in Tshwane, with most fee-paying schools in the district reaching the 80% VU target. The pooled estimate of VU in no-fee schools was 68.8% compared with 78.8% in fee-paying schools, which is comparable to the findings of studies conducted in HICs and LMICs that show that socio-economic status and maternal education may adversely impact VU.^16,17^ School fee-paying status is linked to socio-economic status and maternal education; failure to understand and sign consent for receipt of HPV may lead to nonvaccination. In addition, a lack of awareness because of inadequate access to electronic media such as television and social media may have played a role in the poor VU among girls attending no-fee schools. In some high-income settings, such as North America and Europe, higher maternal education and socio-economic status were associated with lower VU, however.^23,24,25^

Higher VU in Subdistricts 1, 4 and 6 may be attributable to high population density, particularly in townships. These findings are similar to those that show that residing in an urban setting is associated with willingness to vaccinate against HPV.^26^ Furthermore, better access to health promotion materials may have influenced these findings.^25^

The proportion of girls not vaccinated because of caregivers failing to sign consent forms was high. This could be as a result of complicated consent forms^20^ or inadequate social marketing and sensitisation activities. Poor media penetration^20^ by the HPV vaccination programme and negative social media posts may lead to poor VU, as shown in studies in SSA and elsewhere.^27,28^ In addition, the role of vaccine hesitancy cannot be discounted. In a review by Dubé et al., factors such as a lack of knowledge and information were associated with vaccine hesitancy and poor VU.^29^ Leveraging the assistance of community health workers (CHWs) who reside in the subdistricts to increase knowledge and information may help to obtain informed consent from caregivers.^30^ Improving communication and simplifying and streamlining how consent is obtained are strategies recommended in the South African context.^19,20^ It is notable that the missed consent rates declined appreciably between the first and second vaccination campaigns in 2019 (13.6% to 0.3%), which indicates that caregivers were likely more amenable to their children being vaccinated through prior experience with the programme.

School absenteeism contributed to a small proportion of missed vaccinations in the first and second vaccination windows in 2019 (1.2% and 1.1%, respectively). In previous studies from SSA, VU in the school-based HPV vaccination programme was affected by absenteeism.^16^ Improved communication, social marketing and targeted messaging in the weeks leading to the campaign would be expected to reduce absenteeism.

A small proportion of girls (1.2% and 0.4% in the first and second campaigns) were under nine and therefore not eligible to receive HPV. Targeting the 9–14 years age cohort, irrespective of the school grade, may help reduce the number of girls under nine during the HPV vaccination campaign.

### Limitations of the study

This study was a retrospective record review, with possible errors because of missing or incomplete records when the data were recorded. The small number of schools in some subdistricts may have impacted representativeness of the data. Factors not investigated, such as caregiver vaccine hesitancy, educational level and religious or conscientious objections to vaccination, may have confounded the results. The relative effectiveness of uptake of vaccination through school-based compared with health facility–based administration of HPV could not be appraised. Finally, the study was limited to the Tshwane Health District in Gauteng and may not be generalisable to other contexts within South Africa or SSA.

## Conclusion

The authors describe lower than expected HPV vaccination coverage in Tshwane District, South Africa, although schools with a fee-paying structure generally had higher VU than those with a no-fee status. Tshwane Health District should intensify its social mobilisation and sensitisation efforts to help improve VU. Further studies are needed to assess other factors that may impact VU.
